# Case Report: A case of hepatocellular carcinoma with aberrant right hepatic artery treated with transarterial chemoembolization and infusion chemotherapy separately to bilobar lesion combining with systemic therapies and sequential hepatectomy

**DOI:** 10.3389/fonc.2023.1165538

**Published:** 2023-07-04

**Authors:** Yong-Guang Wei, Hao Su, Zi-li Lv, Xi-Wen Liao, Zhi-Ming Zeng, Yu-Xuan Jia, Hua-Sheng Huang, Xiao-Qiang Shen, Guang-Zhi Zhu, Chuang-Ye Han, Xin-Ping Ye, Tao Peng

**Affiliations:** ^1^ Department of Hepatobiliary Surgery, The First Affiliated Hospital of Guangxi Medical University, Nanning, China; ^2^ Guangxi Key Laboratory of Enhanced Recovery after Surgery for Gastrointestinal Cancer, Guangxi Medical University, Nanning, China; ^3^ Department of Pathology, The First Affiliated Hospital of Guangxi Medical University, Nanning, China; ^4^ Department of Oncology, The First Affiliated Hospital of Guangxi Medical University, Nanning, China

**Keywords:** hepatocellular carcinoma, conversion therapy, interventional therapy, hepatic artery variation, case report

## Abstract

**Background:**

Hepatocellular carcinoma (HCC) with a dismal prognosis is the second most deadly malignancy globally. Surgery is believed to be a curative approach. Nevertheless, there is still a considerable probability of postoperative recurrence. Most patients present in advanced stages with a surgically and oncologically unresectable disease. Systemic medicines are increasingly important to downstage the disease and further improve survival.

**Case summary:**

A 67-year-old Chinese man with uncontrolled hepatitis B was discovered to have liver masses with abnormal serum vitamin K absence or antagonist-II (PIVKA-II) level during checkup for upper abdominal discomfort. Abdominal multiphase computerized tomography (CT) and gadoxetate disodium–enhanced magnetic resonance imaging (MRI) showed the bulky bilobar HCCs of Barcelona Clinic Liver Cancer stage B and China Liver Cancer Staging stage IIa. Furthermore, the aberrant right hepatic artery (RHA) originates from the superior mesenteric artery. Due to the location being adjacent to important vasculatures and massive size of the right-sided lesion, curative resection appears to be challenging. To achieve a favorable surgical margin, repeated hepatic arterial infusion chemotherapy (HAIC) was adopted through the variant RHA, while transarterial chemoembolization (TACE) was delivered to the left lobe to arrest tumor growth. Furthermore, sintilimab plus lenvatinib served as the sequential systemic therapy. After 5 months of conversion treatment, the partial response with a decreased serum PIVKA-II level was attained. The R0 hepatectomy was then performed without postoperative complications. The immunohistochemistry and next-generation sequencing results suggested that the two-side HCCs existing tumor heterogeneity were not completely consistent. The patient continues to be without evidence of disease.

**Conclusion:**

Our case highlights a favorable outcome in a man with bilobar bulky HCC after undergoing the comprehensive therapeutic schedule that includes personalized intervention and systemic drug therapy. In terms of conversion therapy, our case provides a secure and practical reference for managing unresectable bilobar HCC coexisting with the aberrant hepatic artery.

## Highlights

- Only 20%–30% of individuals with newly diagnosed early-stage hepatocellular carcinoma (HCC) are amenable to surgical resection, and the recurrence rate at 5 years after surgery is high.
- We experienced a case of bilobar bulky HCCs with right hepatic artery variation.
- Due to the location being adjacent to important vasculatures and size of the right-sided lesion, a multidisciplinary team approach helps to coordinate the personalized conversion treatments—repeated hepatic arterial infusion chemotherapy to the right-side lesion, transarterial chemoembolization to the left lobe, and immunotherapy for systematic treatment.
- Finally, two HCC lesions both achieved partial response and then, a curative hepatectomy was successfully performed.
- The immunohistochemistry and next-generation sequencing results indicated that two-side lesions may be heterogeneous.
- Indeed, for surgically and oncologically unresectable HCC, it is a challenging disease requiring a synthetic and personalized treatment scheme that contributes to controlling tumor progression for a better surgery effect and lower recurrence rate.

## Introduction

Hepatocellular carcinoma (HCC) accounts for 85%–90% of primary liver cancer cases worldwide and is a prevalent malignant tumor of the digestive system (1, 2). The problem is even worse in China. HCC is the fourth most common malignant tumor and the second leading cause of tumor-related death (3, 4). Etiologically, chronic hepatitis B/C virus infection bein prevalent worldwide (5) is one of the vital carcinogenesis factors of the disease (6, 7). However, since most HCC patients have underlying liver cirrhosis or are already in advanced stages, only 20%–30% of newly diagnosed HCC patients are eligible for surgical curative hepatectomy considered as a curative treatment. Therefore, the current therapeutic pattern should attach importance to active conversion therapy to enable more surgical opportunities for unresectable HCC patients.

The concepts of “neoadjuvant therapy” and “conversion therapy” are important in preoperative adjuvant managements (8). For the early-stage patients with proper liver function reserve, surgery remains the mainstay. However, the corresponding 5-year disease recurrence rate is as high as 40%–70% (1, 2). The integrated conversion therapies may provide surgery opportunity (9, 10), prevent relapse, and improve overall survival (11–15). Immunotherapy, molecularly targeted therapy, and interventional therapy are the main conversion techniques. The recent advances in molecularly targeted therapy and immunotherapy have significantly changed the field of advanced HCC treatment and achieved a groundbreaking discovery (16). These therapies can be utilized simultaneously or in sequence to obtain an optimal tumor control or even pathologic complete response (pCR). However, with the not-low non-response rate and acquired drug-resistance rate, the combination may not always be effective, suggesting the complex correlation between the tumor immune microenvironment and the immune response. Meanwhile, the immune-related adverse reactions might also be problematic. There is a widespread recognition of tumor heterogeneity, including intraindividual and intratumor types. An in-depth understanding of the HCC immune microenvironment may be the key to obtaining curative effectiveness. Hence, it is important to highlight how systemic therapy performs best in the given circumstances (17, 18).

Nowadays, the comprehensive management to HCC is mainstream. The diagnosis and treatment model of multidisciplinary collaboration is crucial to addressing specific problems. The targeted countermeasures can be worked out based on tumor heterogeneity, concrete lesion location, anatomic characteristic, liver function, and physical condition to make patients’ benefit maximized.

## Case presentation

**Figure 1:**
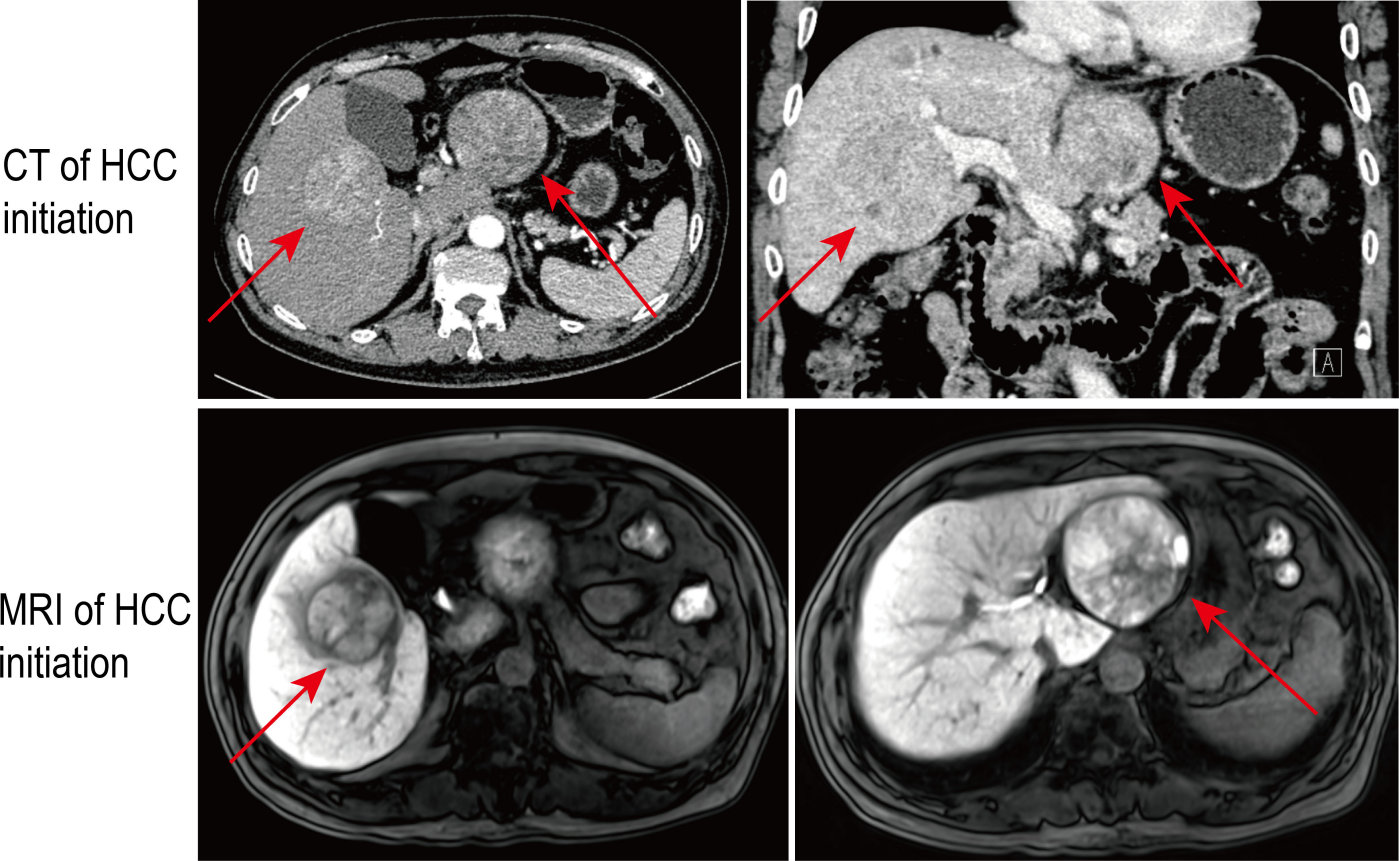
The multiphase liver protocol computed tomography (CT) and gadoxetate disodium–enhanced MRI scan demonstrated the bulky bilobar liver masses with a characteristic radiological appearance of hepatocellular carcinoma (HCC) at first presentation. The red arrows indicate the intrahepatic bilobar tumors.

A 67-year-old male patient was referred to our hospital on 6 April 2022 due to upper abdominal discomfort for half a month. He had a long history of untreated chronic hepatitis B virus (HBV) infection and alcohol use with 200 ml daily intake for 40 years. He reported no other underlying diseases, including hypertension, diabetes, coronary heart disease, and no family history. On physical examination, his body mass index (BMI) was 22.49 kg/m^2^ (weight, 65 kg; height, 170.0 cm). No unremarkable presentations, including ascites and encephalopathy, were detected. A multiphase liver protocol computed tomography (CT) scan demonstrated the bulky bilobar liver masses with a characteristic radiological appearance of HCC (hyperenhancement during the arterial phase and delayed washout in the venous phase)—an exophytic left-lobe lesion measuring 7.2 cm × 7.1 cm × 6.4 cm and a right-lobe lesion measuring 5.6 cm × 5.1 cm × 5.0 cm (**Figure 1**). The diagnosis of bilobar HCCs was further identified by the gadoxetate disodium–enhanced magnetic resonance imaging (MRI) scan and Sonazoid-enhanced contrast-enhanced ultrasound (CEUS). The right hepatic artery (RHA) originating from the superior mesenteric artery (SMA) was noticed. Additionally, intrahepatic multiple dysplasticnodules were also observed in the context of liver cirrhosis and portal hypertension. A sign of mild esophageal varices was revealed by upper gastrointestinal endoscopy. No hepatic or portal venous invasion or thrombosis and the evidence of extrahepatic disease were identified. Using the elastomeric techniques of fibroscan, the measured liver stiffness (LSM) was 14.3 kPa and the controlled attenuation parameter (CAP) indicating moderate hepatic steatosis was 250 dBm. Laboratory tests were as follows: alpha-fetoprotein (AFP), 3.68 ng/ml; protein induced by vitamin K absence or antagonist-II (PIVKA-II), 931.85 mAU/ml; HBV-DNA, 5.56 × 10^5^ IU/ml; total bilirubin, 13.4 μmol/L; albumin, 45.3 g/L; prothrombin time, 13.3 s. Her liver function was well preserved with a calculated indocyanine green retention test after 15 min (ICG-r15) at 12.5%, and the Eastern Cooperative Oncology Group (ECOG) performance status score was Grade 0. A three-dimensional liver model (**Supplementary Figure 1**) was constructed to observe the lesions intuitively and evaluate the liver volume (calculated using the West China formula and the IQQA-Liver system).

## Multidisciplinary expert conclusion

To ensure optimal management, a multidisciplinary team approach was warranted. Major diagnosis was defined—HCC [S5/8 and S2/3, Child–Pugh grade A, China Liver Cancer Staging stage IIa, Barcelona Clinic Liver Cancer (BCLC) stage B]. Given the large size and unfavorable anatomical location being adjacent to the important vasculature of the right-side tumor, an aggressive surgery for the first option may be difficult to obtain a favorable incisal margin and be more likely to recur after surgery. Hence, we should take preoperative adjuvant managements into account. Compared with transarterial chemoembolization (TACE), HAIC could be well behaved at the tumor regression to further achieve a more satisfactory surgical margin (6, 19). In the future, a prospective anatomical hepatolobectomy will be considered for the left disease. Therefore, the semiquantitative conventional TACE could help to control tumor burden (20). Moreover, sequential systemic treatments could contribute to enhancing the conversion effect (21).

## Treatment

**Figure 2:**
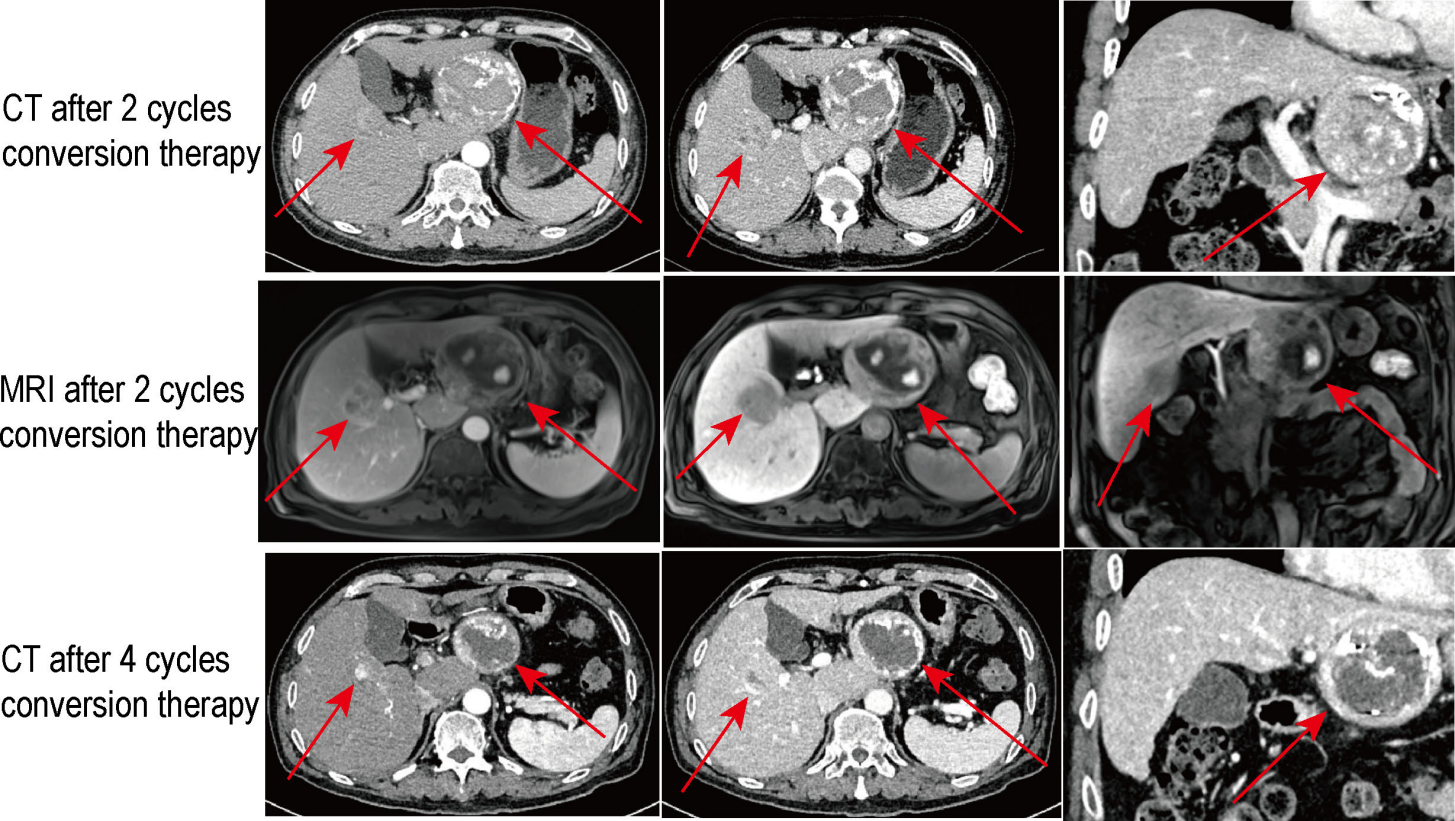
The multiphase CT and gadoxetate disodium–enhanced MRI scan results for review after the conversion therapies of various periods. The red arrows indicate the intrahepatic bilobar tumors.

**Figure 3:**
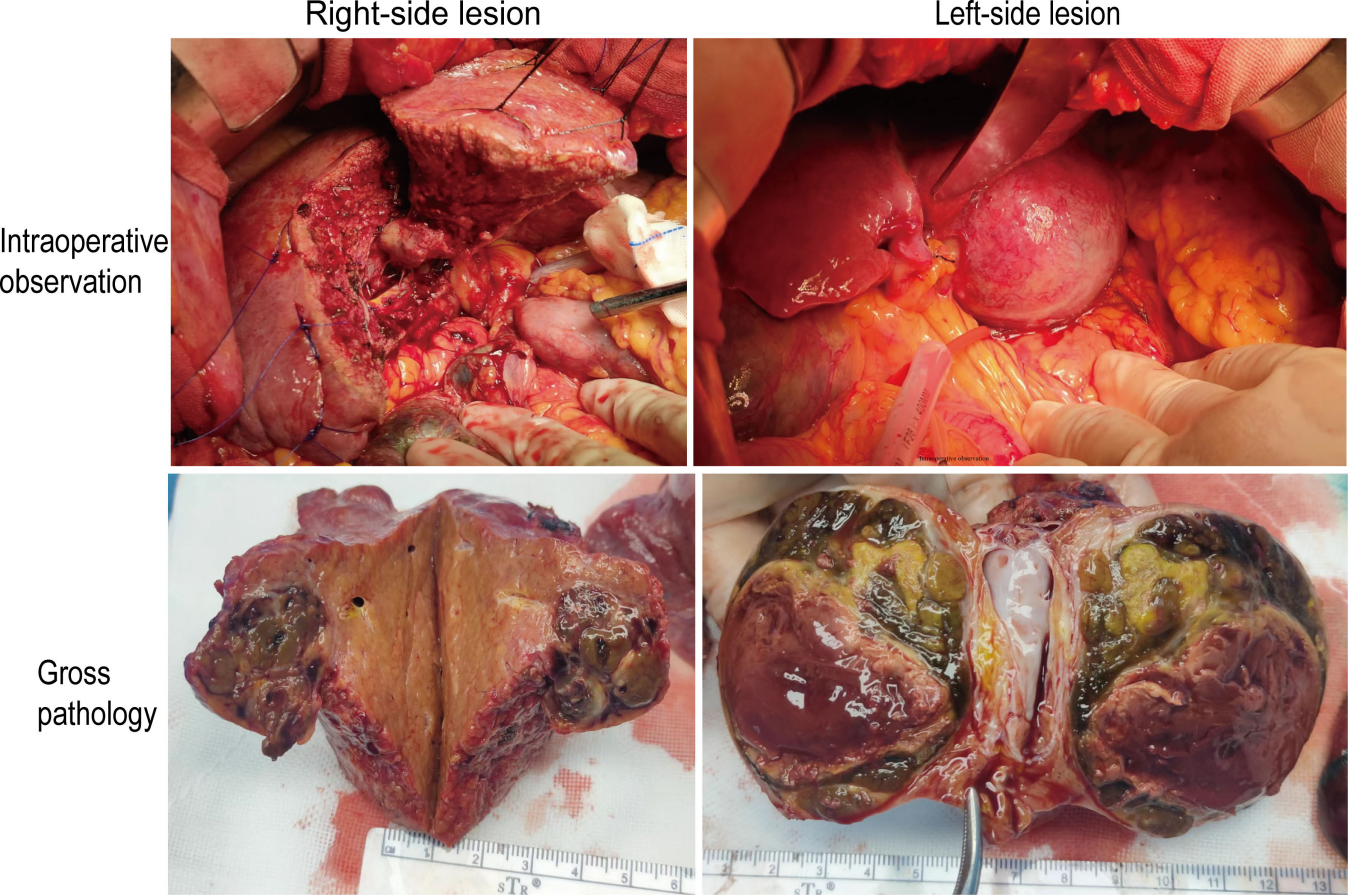
The intraoperative observation and gross pathology of resected specimens.

Firstly, locoregional interventional therapy was carried out. Digital subtraction angiography (DSA) confirmed the variation of the RHA. The sign of additional lesion or arteriovenous fistula was not discovered (**Supplementary Figure 2**). Then, HAIC using the FOLFOX regimen (oxaliplatin 130 mg/m^2^, leucovorin 400 mg/m^2^, fluorouracil bolus 400 mg/m^2^ on day 1, and fluorouracil infusion 2,400 mg/m^2^ for 24 h, Q3W) to the right liver lobe and semiquantitative conventional TACE (cTACE) with emulsions of Lipiodol (5 cc) and local chemotherapy of doxorubicin (20 mg), 5-fluorouracil (500 mg) and cis-platinum (50 mg) at the level of left hepatic artery were adopted synchronously. After the second hospitalization, the patient initiated to use sintilimab injection (200 mg; Q3W) and oral lenvatinib (8 mg; QD) and supplemented with anti-HBV administration (oral entecavir; 0.5 mg; QD) and liver protection therapy. After four cycles of conversion treatments, the abdominal contrast-enhanced CT for review showed the partial responses (PRs) of the bilobar tumor based on the modified Response Evaluation Criteria in Solid Tumors criterion (22). The signs of the shrank lesions, partial tumor necrosis, and slight intralesion bleeding were discovered (**Figure 2**). Accordingly, the serum PIVKA-II had significantly decreased while the peripheral blood CD4^+^ and CD8^+^ T lymphocyte increased from previous. With the ICG-r15 for review being 17.9%, he was referred for hepatectomy. For a surgeon, the case was quite challenging because the right-side lesion being a next neighbor to the trunk of right hepatic vein was at the junction region of the right anterior and right posterior portal vein (**Figure 3**). A radical and safe operation was required to not only ensure a negative margin successfully but also preserve the surrounding important vasculature. After a month off sintilimab, the resection of right-sided lesion and the formal left lobectomy were performed successfully with the assistance of a preoperative 3D model (**Supplementary Figure 3**) and intraoperative ultrasound that helped to determine the resection margin. Furthermore, a novel bile leakage detecting approach (Peng’s test) was adopted to reduce the risk of postoperative complications (23).

## Outcome and follow-up

**Figure 4:**
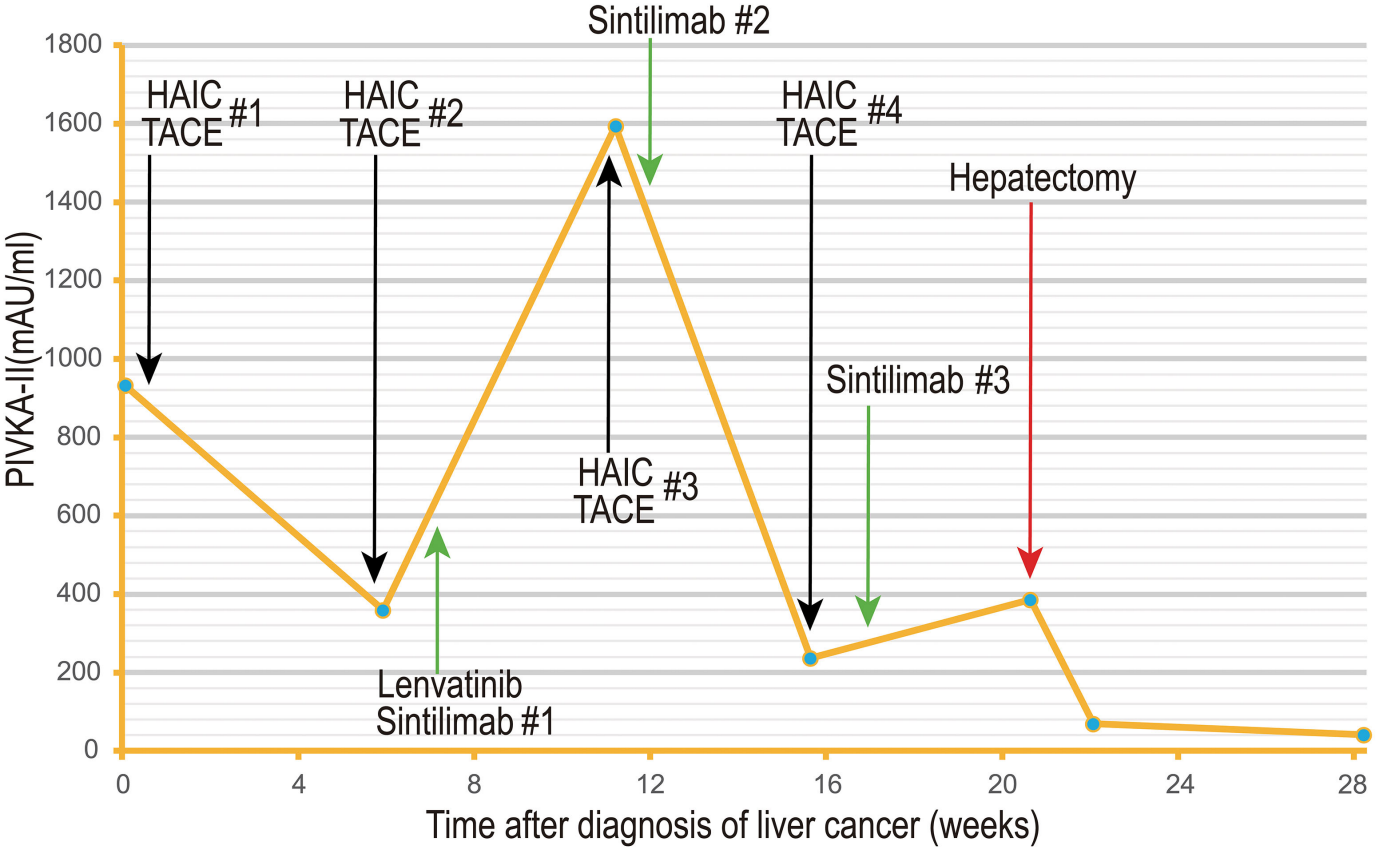
The serum PIVKA-II level (mAU/ml) had decreased significantly during the treatment course. The number after the pound sign ("#") represents the cycle of a form of treatment.

The gross pathology of the resected specimens showed the right lesion measuring 2.5 cm × 2.5 cm × 2.5 cm with approximately 40% tumor necrosis and the left lesion measuring 6.0 cm × 6.0 cm × 5.0 cm with approximately 50% tumor necrosis were both intact and unbroken. The well-defined border and peripheral pseudocapsule were found (**Figure 3**). No residual tumor tissue, definite vascular carcinoma thrombi, and satellite nodules were observed. Microscopically, the specimens accorded with Edmondson–Steiner differentiation II grade HCC without any microvascular invasion (MVI). The fibrous capsule and intratumor fibrous tissue hyperplasia were also observed in the lesion. The negative surgical margin was confirmed. Additionally, The immunohistochemistry (IHC) and next-generation sequencing (NGS) comprehensive panel were conducted (**Tables 1**, **2**). The HBsAg-positive expression indicated the HBV infection of para-carcinoma liver tissue. Both lesions showed the negative programmed death 1 (PD-1)/Programmed cell death-Ligand 1 (PD-L1) expressions (**Supplementary Figure 4**) and the low tumor mutational burden (TMB)—less than 20 mutations per megabase. The comprehensive results suggested that the two HCCs may be heterogeneous. Consequently, the patient was discharged on the eighth postoperative day without postoperative complications. His serum AFP stayed negative during the treatment (**Supplementary Figure 5**). His serum PIVKA-II had decreased significantly at the postoperative review (**Figure 4**), and he continues to be without evidence of disease.

**Table 1:** Representative immunohistochemistry of the two-side tumor tissues.

	Left-side lesion	Right-side lesion
Arginase-1	Focally positive	Positive
Glypican-3	Partly positive	Negative
Hepatocyte	Positive	Positive
CD34*	Positive	Positive
*VEGF*	Weakly positive	Negative
*CK19*	Negative	Negative
*CK7*	Negative	Negative
*NM23*	Positive	Negative
*P21*	Negative	Negative
*P53*	Weakly positive	Negative
*PD-1/PD-L1*	Negative	Negative
*Ki-67*	2%	8%

*The pattern of CD34 expression (higher than 50 items per high power field) indicates the occurrence of capillary vascularization.

**Table 2:** The results of next-generation sequencing detection including the actionable mutation genes and abundance.

Left-side lesion		Right-side lesion	
Genes	Abundance	Genes	Abundance
*TP53*	45.1%	*KMT2C*	28.0%
*CTNNB1*	36.0%	*CTNNB1*	15.2%
*KDR*	41.6%	*KDR*	50.0%
*ARID1B*	48.6%	*ARID1B*	22.8%
*ARID1A*	66.5%	*ESR1*	53.6%
*BRCA2*	52.0%		
*SRSF2*	49.3%		
*PTEN*	45.2%		
*POLE*	45.9%		
*EPHB1*	25.0%		
*GATA1*	100.0%		

TP53, tumor protein p53; CTNNB1, catenin beta 1; KDR; kinase insert domain receptor; ARID1B, AT-rich interaction domain 1B; ARID1A, AT-rich interaction domain 1A; BRCA2, BRCA2 DNA repair associated; SRSF2, serine and arginine rich splicing factor 2; PTEN, phosphatase and tensin homolog; POLE, DNA polymerase epsilon, catalytic subunit; EPHB1, EPH receptor B1; GATA1, GATA binding protein 1; KMT2C, lysine methyltransferase 2C; ESR1, estrogen receptor 1.

## Discussion

Surgical treatment alone is beneficial for patients in the early stage or partial middle stage. However, a high recurrence rate may frustrate our confidence. Notwithstanding, studies have confirmed the efficacy of hepatic resection for the HCC patients at BCLC stage B or C (24, 25), aggressive surgery may not result in a better relapse-free or overall survival compared to systemic therapy (26–28). In recent years, a novel and debatable concept, “oncologically unresectable disease,” was put forward. It speaks of HCCs that can be surgically removed. However, the survival outcomes of surgery are not always better than those of non-operative therapy. It is challenging to completely remove the tumor and perform a clinically curative resection if the resection margin is adjacent to crucial vessels or ducts. Even without vascular invasion or extrahepatic dissemination, patients with large multifocal HCC afflicting both lobes correspond to a more advanced tumor stage due to the substantial tumor load, rather than a subclass of intermediate stage (29). In this case, multiple tumors, initial active hepatitis, and a potentially positive surgical margin all increase the probability factors of postoperative recurrence (29, 30). Hence, a comprehensive antineoplastic protocol is more advocated. Tumor downstaging can be achieved using multiple approaches in a combined or sequential manner. In our case, the initially local resection may be not superior to the preoperative adjuvant treatments. Combined with a sequential *PD-1* inhibitor and molecularly targeted drugs, the combined HAIC and TACE to the different liver lobes appeared to be a safe and effective conversion modality for the bilobar HCC.

Remarkably, the lag period between conversion therapy and surgery may increase the risk of tumor progression. Few studies prior established the duration of drug withdrawal of systemic treatment before surgery. Some researchers recommended that conversion surgery should be performed within 4 weeks after the final immunotherapy cycle (31–33). To avoid missing the ideal timing for surgery, the treatment effects on the tumor and liver function reserve should be evaluated in a short period. After surgery, there is a dearth of high-level and evidence-based research on whether the adjuvant therapy is still necessary to avoid the potential recurrence after surgery. In any case, the routine follow-ups are supposed to be continued.

Serving as a bridging and downstaging treatment modality, trans-arterial interventional therapy causes the release of intratumoral inflammatory medium and neotigens and further induces migration of T cells and then improves the effect of immunotherapy and molecularly targeted therapy (20, 34). It is well recognized that TACE is a cornerstone therapy with a considerable success rate in the treatments of advanced HCC (35). Moreover, when compared to other imaging modalities, DSA can reveal undetected lesions early on (36). In the perioperative HCC treatment, HAIC with the FOLFOX regimen has attracted extensive attention (15, 37). In a multicenter clinical trial, FOLFOX-HAIC had a greater surgical conversion rate than TACE and offered unresectable HCC patients a better prognosis (38). In terms of tumor regression, the repeated FOLFOX-HAICs provide more advantages, which is more aligned with the requirements of reserving a more normal liver and ameliorating the surgical margin (6). Comparatively speaking, TACE, which causes the necrosis of cancer cells, is preferable for reducing tumor load. In fact, large-sample, prospective, and randomized controlled studies are still needed to compare the therapeutic efficacy of TACE and HAIC.

According to a study, aggressive hepatic resection combined with radiofrequency ablation in treating proper patients with bilobar HCC could offer long-term survival, which was rarely achieved by TACE. In a single-center retrospective study targeting the bilobar HCC with or without portal vein thrombosis, transarterial radioembolization was delivered to the hepatic lobe with large HCC, while TACE was administered to another lobe with minor HCC(s). Such a therapeutic schedule has a good effect without increasing complications (39). Anatomically, aberrant RHA mainly originates from the SMA (40, 41). One of the characteristics of HAIC is continuous local administration. It makes sense to maintain uniform anticancer medication distribution throughout the entire tumor to carry out repeated HAIC effectively (42). In our case, given the RHA variation, the traditional common hepatic artery catheterization cannot take into account the tumors in both lobes. Nevertheless, the dual-port approach is a feasible technique to address such a conundrum without the hemodynamic modification of anatomic hepatic artery variation (43).

Lenvatinib is a representative multikinase inhibitor. As the first-line treatment of advanced unresectable HCC patients, it has emerged as a desirable alternative (44). Moreover, the emerging immunotherapies including the immune checkpoint inhibitor (ICI), T-cell transfer therapy, monoclonal antibody, treatment vaccine, immune system modulator, dendritic cell–based immunotherapy, and antibody–drug conjugate are demonstrated to be the essential tumor treatment modality both today and in the future (45). Especially, ICIs targeting the interaction between T cells and tumor cells have received groundbreaking progress. However, the overall response rates (ORRs) of monotherapy with either singular immunotherapy or molecularly targeted agent alone leave much to be desired. Consociation therapy with the higher ORRs would be mainstream. It has been shown that combining tyrosine kinase inhibitors with ICIs can achieve the synergistic effect to overcome the immunosuppressive nature of the tumor microenvironment (27, 46). Lenvatinib also shown improved anticancer efficacy when coupled with an anti-PD-1 antibody (47–49). However, the managements of toxicity associated with perioperative systemic therapy should be highly regarded. Thereinto, the presence of immune hepatitis increases the perioperative death risk. In our case, the patient carried the chronic HBV infection and liver cirrhosis with portal hypertension; thus, the antiviral action should be expected to be emphasized during the periodic systemic therapy to refrain from HBV reactivation (50).

Given the complex heterogeneity, the conversion strategies of bulky bilobar HCCs that are most effective probably depends on the individual. When faced with complex first- and second-line options for systemic treatment with not low acquired drug resistance, the preoperative puncture of targeted disease under ultrasound guidance can obtain the original cancerous tissue for NGS, IHC, and immune-infiltration detection to describe the driven genes and molecular alterations possibly (51–53). However, the risk of needle tract implantation metastasis may be ponderable trouble. Most prevalent gene mutations, such as *TERT*, *TP53*, or *CTNNB1*, are not actionable. Only approximately 25% of HCCs contain actionable mutations (54). Carla Montironi (55) et al. have described the immunogenomic contexture of HCC that could be defined as the inflamed and non-inflamed tumors (56). The inflamed immunogenic tumor that has substantial activated immune cells in the tumor microenvironment is more likely to react to systemic therapy. Additionally, the degree of *PD-1/PD-L1* expression in a tumor is connected to the clinical effectiveness of anti-*PD-1/PD-L1* therapeutics. Nevertheless, the tumor with a negative expression should not be entirely ruled out (33).

## Conclusion

To maximize the patient’s benefit, a comprehensive and individualized treatment scheme should be developed for surgically and oncologically unresectable HCC. The preoperative repeated FOLFOX-HAIC has a favorable effect on tumor shrinkage and may improve the operable rate.

## Data availability statement

The datasets presented in this study can be found online at: https://ngdc.cncb.ac.cn/gsa-human/. The accession number is HRA004924.

## Ethics statement

The studies involving human participants were reviewed and approved by Medical Ethics Committee of the First Affiliated Hospital of Guangxi Medical University (Approval Number: 2023-E161-01). The patients/participants provided their written informed consent to participate in this study. All procedures performed in this study were in accordance with the ethical standards of the institutional and/or national research committee(s) and with the Helsinki Declaration. Informed written consent was obtained from the patient for publication of this report and any relevant images.

## Author contributions

TP and HS designed the report; Y-GW and Y-XJ were involved in the drafting of the manuscript; H-SH, Z-MZ, and X-WL were involved in the collation of clinical data and radiological images. G-ZZ, C-YH, Z-MZ, and X-PY were involved in the manuscript revision. Z-LL contributed to pathological examination. All authors contributed to the article and approved the submitted version.

## Supplementary material

Supplementary Figure 1 The preliminary 3D liver model. A red arrow indicates the aberrant right hepatic artery (RHA) originating from superior mesenteric artery (SMA).

Supplementary Figure 2 Interventional therapy. A: The arteriography of right hepatic artery originating from superior mesenteric artery; B: TACE in the left lobe through the left hepatic artery and abdominal trunk; C: The third arteriography representing the intra-tumoral lipiodol deposition after repeated TACE to the left lobe.

Supplementary Figure 3 The reconstructed 3D liver model before surgery.

Supplementary Figure 4 Immunohistochemistry (IHC), including H&E and *PD-1/PD-L1* staining, of representative pathological sections. *PD-1*, Programmed death-1; *PD-L1*, Programmed cell death-Ligand 1.

Supplementary Figure 5 The serum AFP level (ng/ml) stayed negative during the treatment course. The number after the pound sign ("#") represents the cycle of a form of treatment.
