# Supplementary figures and images for: Case Report: A case of hepatocellular carcinoma with aberrant right hepatic artery treated with transarterial chemoembolization and infusion chemotherapy separately to bilobar lesion combining with systemic therapies and sequential hepatectomy
Source: Front Oncol. 2023 Jul 4;13:1165538. doi: 10.3389/fonc.2023.1165538 (PMC10353483; doi:10.3389/fonc.2023.1165538)

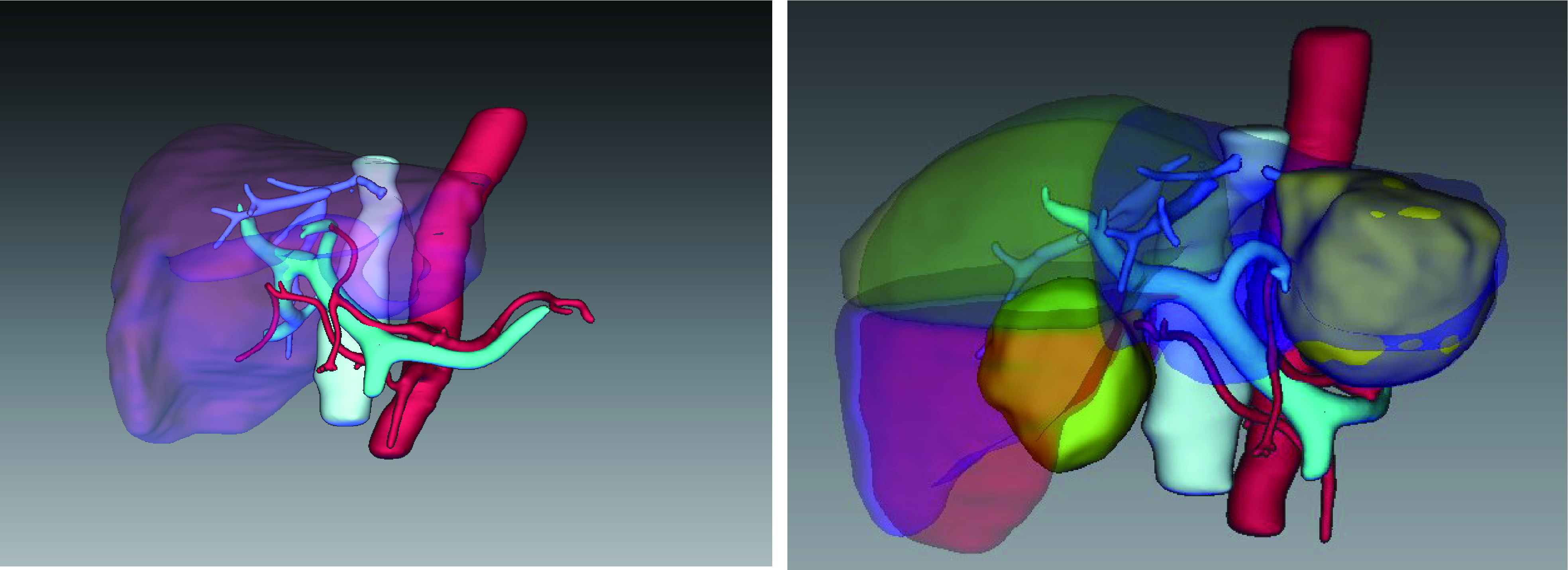

Supplement: Supplementary Figure 1 — The preliminary 3D liver model. A red arrow indicates the aberrant right hepatic artery (RHA) originating from superior mesenteric artery (SMA). [file Image_1.tif]

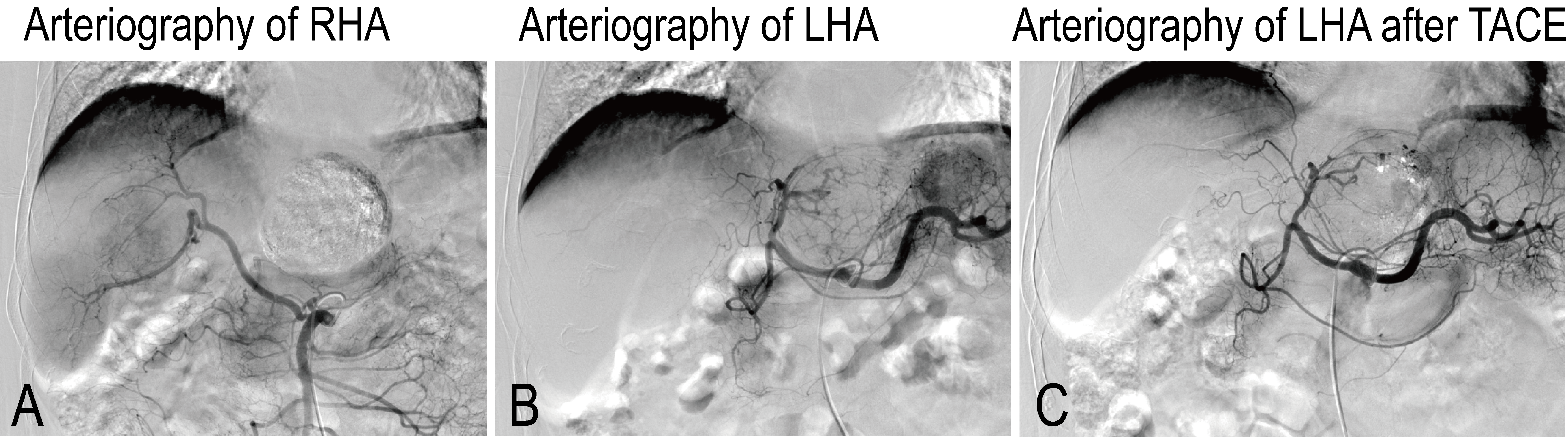

Supplement: Supplementary Figure 2 — Interventional therapy. A: The arteriography of right hepatic artery originating from superior mesenteric artery; B: TACE in the left lobe through the left hepatic artery and abdominal trunk; C: The third arteriography representing the intra-tumoral lipiodol deposition after repeated TACE to the left lobe. [file Image_2.tif]

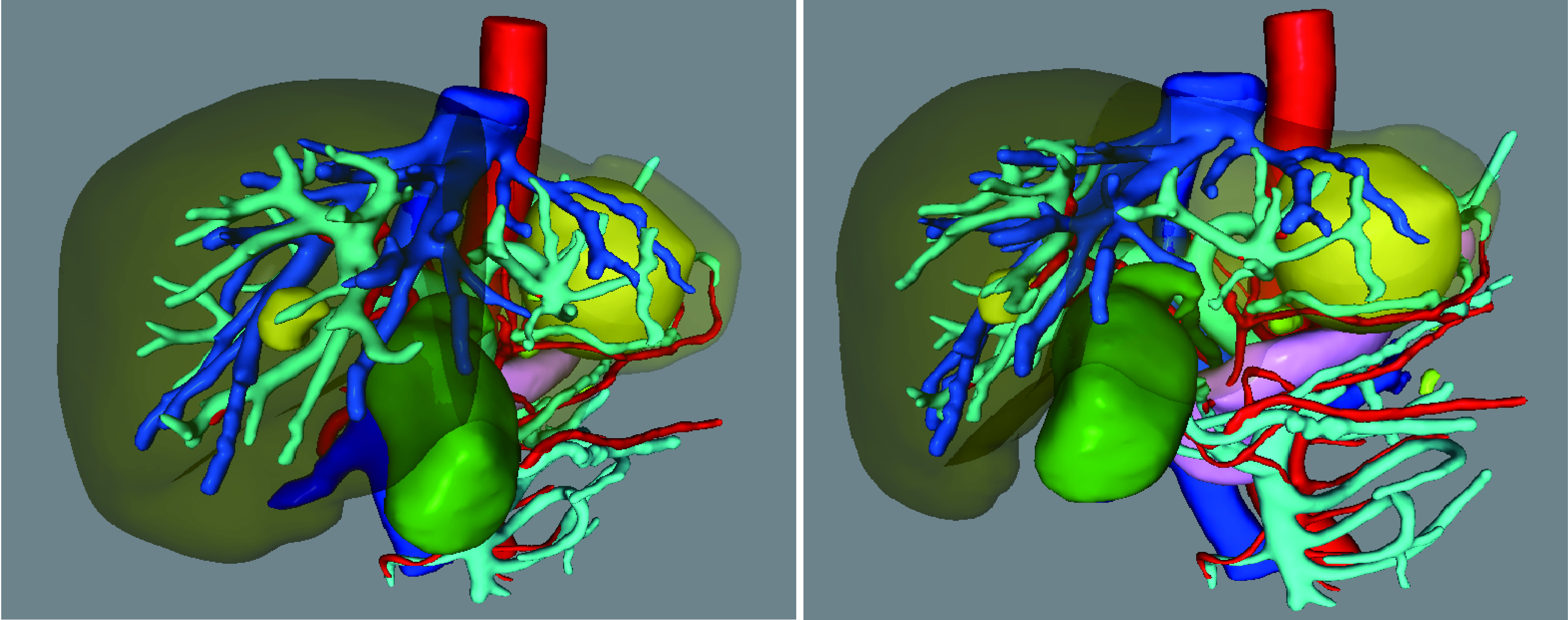

Supplement: Supplementary Figure 3 — The reconstructed 3D liver model before surgery. [file Image_3.tif]

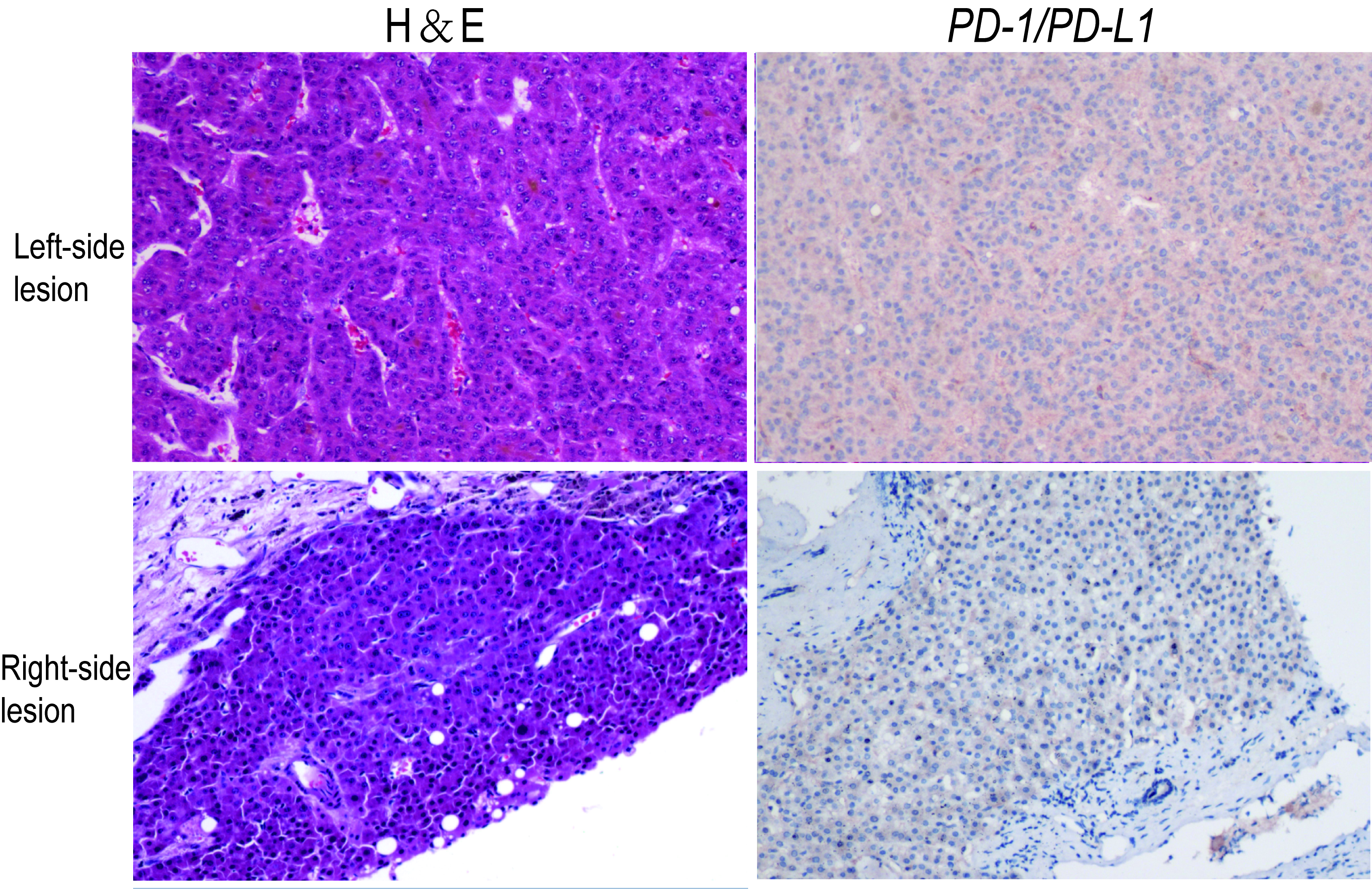

Supplement: Supplementary Figure 4 — Immunohistochemistry (IHC), including H&E and PD-1/PD-L1 staining, of representative pathological sections. PD-1, Programmed death-1; PD-L1, Programmed cell death-Ligand 1. [file Image_4.tif]

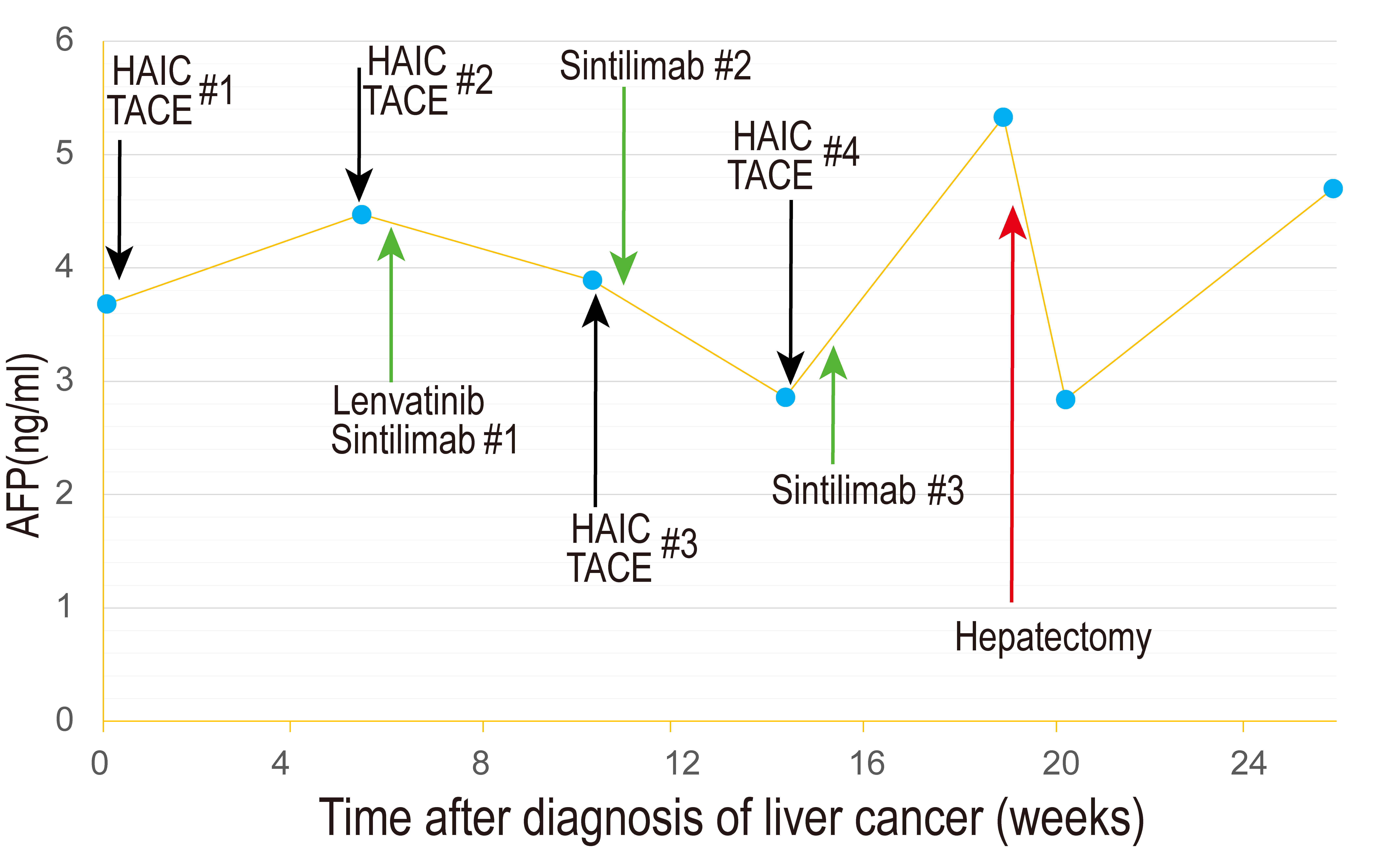

Supplement: Supplementary Figure 5 — The serum AFP level (ng/ml) stayed negative during the treatment course. The number after the pound sign ("#") represents the cycle of a form of treatment. [file Image_5.tif]
